# X-Chromosomal Maternal and Fetal SNPs and the Risk of Spontaneous Preterm Delivery in a Danish/Norwegian Genome-Wide Association Study

**DOI:** 10.1371/journal.pone.0061781

**Published:** 2013-04-16

**Authors:** Solveig Myking, Heather A. Boyd, Ronny Myhre, Bjarke Feenstra, Astanand Jugessur, Aase S. Devold Pay, Ingrid H. G. Østensen, Nils-Halvdan Morken, Tamara Busch, Kelli K. Ryckman, Frank Geller, Per Magnus, Håkon K. Gjessing, Mads Melbye, Bo Jacobsson, Jeffrey C. Murray

**Affiliations:** 1 Department of Genes and Environment, Division of Epidemiology, Norwegian Institute of Public Health, Oslo, Norway; 2 Department of Epidemiology Research, Statens Serum Institut, Copenhagen, Denmark; 3 Craniofacial Research, Murdoch Childrens Research Institute, Royal Children’s Hospital, Parkville, Australia; 4 Department of Obstetrics and Gynecology, Women and Children’s Division, Oslo University Hospital, Oslo, Norway; 5 Department of Public Health and Primary Health Care, University of Bergen, Bergen, Norway; 6 Department of Obstetrics and Gynecology, Haukeland University Hospital, Bergen, Norway; 7 Department of Obstetrics and Gynecology, Institute for the Health of Women and Children, Sahlgrenska University Hospital, Göteborg, Sweden; 8 Department of Pediatrics, Carver College of Medicine, University of Iowa, Iowa City, Iowa, United States of America; 9 Department of Epidemiology, College of Public Health, University of Iowa, Iowa City, Iowa, United States of America; University of Nevada School of Medicine, United States of America

## Abstract

**Background:**

Recent epidemiological studies suggest that the maternal genome is an important contributor to spontaneous preterm delivery (PTD). There is also a significant excess of males among preterm born infants, which may imply an X-linked mode of inheritance for a subset of cases. To explore this, we examined the effect of maternal and fetal X-chromosomal single nucleotide polymorphisms (SNPs) on the risk of PTD in two independent genome-wide association studies and one replication study.

**Methods:**

Participants were recruited from the Danish National Birth Cohort and the Norwegian Mother and Child cohort studies. Data from these two populations were first analyzed independently, and then combined in a meta-analysis. Overall, we evaluated 12,211 SNPs in 1,535 case-mother dyads and 1,487 control-mother dyads. Analyses were done using a hybrid design that combines case-mother dyads and control-mother dyads, as implemented in the Haplin statistical software package. A sex-stratified analysis was performed for the fetal SNPs. In the replication study, 10 maternal and 16 fetal SNPs were analyzed using case-parent triads from independent studies of PTD in the United States, Argentina and Denmark.

**Results:**

In the meta-analysis, the G allele at the maternal SNP rs2747022 in the FERM domain containing 7 gene (*FRMD7*) increased the risk of spontaneous PTD by 1.2 (95% confidence interval (CI): 1.1, 1.4). Although an association with this SNP was confirmed in the replication study, it was no longer statistically significant after a Bonferroni correction for multiple testing.

**Conclusion:**

We did not find strong evidence in our data to implicate X-chromosomal SNPs in the etiology of spontaneous PTD. Although non-significant after correction for multiple testing, the mother’s G allele at rs2747022 in *FRMD7* increased the risk of spontaneous PTD across all populations in this study, thus warranting further investigation in other populations.

## Introduction

Preterm delivery (PTD), defined as delivery before 37 weeks of gestation, affected 15 million births in 2010 [1]. It is associated with a substantially increased risk of mortality, as well as short- and long-term morbidity [1], [2]. The PTD rate ranges from 6% in Scandinavian countries [3] to 18% in some African populations [1]. PTD is routinely divided into two main groups according to clinical presentation: i) *spontaneous PTD*, in which delivery starts with either uterine contractions (preterm labor) or membrane rupture (preterm prelabor rupture of membranes (PPROM)), and ii) *iatrogenic PTD*, which is induced by medical or surgical intervention.

Spontaneous PTD is etiologically heterogeneous, involving both genetic and environmental risk factors. Twin studies have estimated the heritability of PTD at 17–36% [4], [5], and that of parturition timing at 34% [6]. Although there is compelling evidence for a genetic component to PTD, no common genetic variants or mode of inheritance have yet been established [7]. Recent generational epidemiological studies suggest that the maternal genome is a key genetic contributor to PTD inheritance [7]–[9], and a personal history of PTD is considered the most important risk factor for PTD in multiparous women [7]. Women who were born preterm themselves, or who have sisters or maternal half-sisters with a history of PTD, are also at increased risk [7], [8].

A small but significant excess of males has been observed among preterm-born infants in most populations, especially for spontaneous PTD [10]. Different hypotheses have been proposed to explain this excess, including a shorter gestational length due to higher average fetal weight [11], increased vulnerability to certain pregnancy complications [10], and the fact that biochemical processes, such as increased estrogen production from androgen precursors or higher levels of interleukin-1 in the amniotic fluid of males, could lead to uterine contractions and PTD [12], [13]. It is also possible that this male excess is due to fetal X-linked risk alleles contributing to a subset of the PTD cases, in which hemizygosity would increase the risk compared to heterozygosity [12].

Several complex disorders have been associated with genetic variants on the X chromosome, including prostate cancer [14], type 2 diabetes [15], X-linked dystonia Parkinsonism [16] and psychiatric disorders such as schizophrenia and autism spectrum disorders [17]. Hypospadias, a congenital malformation of male external genitalia, is strongly associated with genetic variants on the X chromosome [18]. As males inherit only one X chromosome, they are hemizygous for X-linked genes, and although females inherit two X chromosomes, one of them is inactivated in each cell. A recent meta-analysis found a significant association between skewed X-chromosome inactivation in females and idiopathic recurrent spontaneous abortion [19]. Recurrent second-trimester spontaneous abortion is a well-known risk factor for spontaneous PTD [20], [21], and it is plausible that some of the same mechanisms may be involved in both outcomes.

Most candidate-gene studies of PTD have used a case-control study design and focused primarily on autosomal markers. This may be partly due to limited knowledge about plausible X-linked candidate genes for PTD and a lack of appropriate statistical methodology for family-based association studies of X chromosome markers [22]. Nearly all methods that have subsequently been developed for handling X-linked markers are based on the transmission/disequilibrium test (TDT) [23]–[27]. A family-based likelihood ratio test for the X chromosome (X-LRT) [28] and Haplin [22] are among the few existing methods that can estimate genetic relative risks, in contrast to other methods [23]–[25], [27], [29] that only generate a p-value for hypothesis-testing.

Taking into consideration the strong evidence of a maternally mediated genetic effect in PTD and the higher proportion of males among infants born preterm, we examined the effects of maternal and fetal X-linked gene variants on the risk of PTD, using data from two genome-wide association studies (GWAS) in Scandinavia (Norway and Denmark). To verify our findings, we conducted a replication study using case-parent triads from independent studies in the United States, Argentina and Denmark.

## Materials and Methods

### Ethics Statement

All the studies outlined in this paper were approved by the regional ethics committees or institutional review boards (IRB) at each site, and a written informed consent was obtained from each participant. The Danish National Birth Cohort (DNBC) study protocol was approved by the Danish Scientific Ethics Committee for the Copenhagen Capital City Region and by the Danish Data Protection Agency. The Norwegian Mother and Child Cohort Study (MoBa) was approved by the Regional Committee for Medical Research Ethics in South-Eastern Norway and the Norwegian Data Inspectorate. For the replication study, ethics approvals were obtained from the Human Subjects Office, University of Iowa (Iowa City, USA), the University of Pittsburgh Institutional Review Board (Pittsburgh Pennsylvania), the University of Rochester Research Subjects Review Board (Rochester, New York), the Wake Forest University Review Board (Wake Forest, North Carolina), and El Comite de Etica en Investigacion del Cemic (Argentina).

### Study Participants

This study was conducted using data from DNBC [30] and MoBa [31]. A replication study of 10 of the most significant maternal SNPs and 16 of the most significant fetal SNPs from the combined analysis of DNBC and MoBa data was performed in independent family studies of PTD in the United States (US), Argentina and Denmark.

#### DNBC

The DNBC includes approximately 100,000 pregnancies from 1996 to 2002. Women were invited to participate at their first prenatal visit with their general practitioner at gestational weeks 6–12 [30]. Information on exposures not registered in medical records was obtained from national registers, telephone interviews, and a food frequency questionnaire. Blood samples from mothers were collected by the general practitioner during the routine visit at gestational weeks 6–12 and 24, and a cord blood sample was taken at delivery. Blood samples were stored in the Danish National Biobank at the Statens Serum Institut in Copenhagen, Denmark.

A case was defined as a live, singleton spontaneous PTD occurring before 259 days (37 weeks) of gestation; a control was defined as a live, singleton full-term delivery (i.e. occurring at 280–286 days (40 weeks) of gestation). The exclusion criteria were: fetal malformations, preeclampsia/eclampsia, placenta previa, placental abruption, polyhydramnios, isoimmunization and placental insufficiency. In addition, the infant’s parents and grandparents had to be of Nordic ancestry (i.e. born in Denmark or one of the other Nordic countries).

#### MoBa

MoBa is a nationwide Norwegian pregnancy cohort study administered by the Norwegian Institute of Public Health (NIPH). The study includes more than 107,000 pregnancies recruited from 1999 through 2008. Women were invited by postal invitation in connection with a routine ultrasound screening offered to all pregnant women in Norway at gestational weeks 17–19. Most of the pregnant women in Norway were invited and the participation rate was 42.7%. Participation rates for the first three questionnaires were 92–95% [31]. For the current study, cases and controls were selected from Version 4 of the MoBa cohort, which included a total of 71,669 pregnancies. This version was released in 2008 for research use.

Blood samples were drawn from the pregnant woman and the fetus’ father during the ultrasound appointment. A new blood sample from the woman and a cord-blood sample from the infant were collected at delivery. All biological specimens were sent to the MoBa Biobank where DNA was extracted, processed and stored until retrieval [32].

A case was defined as a live, singleton spontaneous PTD occurring between 154 and 258 days of gestation (22^0/7^–36^6/7^ weeks); a control was defined as a live, singleton full-term delivery, i.e. occurring at 273–286 days of gestation (39^0/7^ and 40^6/7^ weeks). Gestational age was estimated by ultrasound at gestational weeks 17–19. In the few cases without ultrasound dating, gestational age was estimated using the date of the last menstrual period. Strict selection criteria were applied to both cases and controls in order to yield the clearest possible phenotype. Only women in the age group 20–34 years were selected. As women aged <20 years and >35 years have an increased risk of spontaneous PTD [21], only women in the age group 20–34 years were selected in order to prevent the increased risk from affecting the results. Pregnancies involving pre-existing medical conditions, such as diabetes, hypertension, specific autoimmune diseases (inflammatory bowel disease, systemic lupus erythematosus, rheumatoid arthritis and scleroderma) and immune-compromised conditions, were excluded from the study. Lastly, pregnancies with complications such as preeclampsia, hypertension, gestational diabetes, placental abruption, placenta previa, cervical cerclage, small for gestational age and fetal malformation were also excluded, as were pregnancies conceived by *in vitro* fertilization.

#### The US prematurity study

This study focuses on PTD cases and their families. Study participants (cases and both parents and grandparents when available) were enrolled at various locations in the US, including Iowa City (Iowa), Wake Forest (North Carolina), Pittsburgh (Pennsylvania) and Rochester (New York). DNA from each participant was extracted from saliva or blood samples. Cases were defined as PTD if gestational age was <37 weeks. In order to harmonize the phenotypes of the replication cohort with those of the original study populations, we excluded indicated deliveries without PPROM, multiple gestations, fetal malformations, preeclampsia or hypertension in pregnancy, placental abruption, placenta previa, conception via assisted reproductive technology, and maternal age <20 or >39 years. Only white, non-Hispanic individuals were included in the study. For the maternal replication, all ethnicities were included in one of the analyses, but after other inclusion criteria were applied, only white, non-Hispanic individuals remained for analysis.

#### The argentina prematurity study

The Argentina Prematurity Study had similar enrollment criteria as the US Prematurity study. Cases were defined as PTD if gestational age was <37 weeks. We excluded deliveries with multiple gestations, preeclampsia, maternal age <20 or >39 years, and indicated deliveries without PPROM. For the current study, maternal SNPs were replicated using triads consisting of the mother of a preterm-born infant and her parents.

#### The denmark family study of PTD

This study includes mothers of preterm-born infants and their parents (the infant’s maternal grandparents). Cases were defined as PTD if gestational age was <37 weeks. Indicated deliveries and congenital malformations were excluded.

### Genotyping

The Illumina Human660W-Quad BeadChip platform (Illumina, San Diego, CA, USA) was used for genotyping in both study populations. The DNBC samples were genotyped by the Center for Inherited Disease Research (CIDR) at the Johns Hopkins University (Baltimore, MD, USA). The MoBa samples were genotyped at the genotyping core facility at Oslo University Hospital (Oslo, Norway). For replication, the 28 selected SNPs were genotyped at the University of Iowa using the TaqMan® chemistry genotyping system (Applied Biosystems, Foster City, CA, USA). All reactions were performed under standard conditions supplied by Applied Biosystems. Following thermocycling, fluorescence levels of the FAM and VIC dyes were measured and genotypes were scored using the proprietary Sequence Detection Systems 2.2 software (Applied Biosystems) and reviewed manually by at least two independent observers.

### Quality Control

#### DNBC

In the Danish data, 2,035 mother-infant pairs were available for analysis (1,061 case pairs and 974 control pairs). Of these, 20 dyads were excluded because of a call rate <97% in either the mother or the infant. One dyad was excluded because of unknown gender in the infant, and a further nine dyads were excluded because either the mother or the infant had a sibling or half-sibling in the cohort. After quality control, 2,005 mother-infant dyads (1,046 case dyads and 959 control dyads) were available for the current analysis.

Before data processing, there were 14,441 SNPs on the X chromosome. After filtering out SNPs that had a call rate <95%, more than five Mendelian inconsistencies, or a minor allele frequency (MAF) of <1%, we were left with 12,345 SNPs for further analysis.

#### MoBa

In the Norwegian data, there were 1,086 complete mother-infant dyads eligible for analysis (529 case dyads and 557 control dyads). Sixty-two dyads were excluded because they had a genotype call rate <97% in either the mother or the infant. In addition, 7 mother-infant pairs were excluded because of inconsistencies in parenthood. This left 1,017 mother-infant dyads (489 case-dyads and 528 control-dyads) for the current analysis.

Overall, 14,441 SNPs on the X chromosome were available before data processing. SNPs that had a call rate <95%, more than five Mendelian inconsistencies, or a MAF <1% were excluded, leaving 12,361 SNPs for the current analysis.

#### The US prematurity study and the denmark family study of PTD

The fetal triads consisted of a preterm infant and his/her parents. For the fetal triads, 286 case families (787 individuals) were eligible for analysis. Of these, three families (15 individuals) were removed because of Mendelian inconsistencies, 25 individuals were removed because of a call rate <90%, 8 individuals were subsequently removed because there was only one person left in the family, and six mother-father pairs (12 individuals) were removed because no data/genotypes were available on the infant. This left 267 families (727 individuals) for further analysis. A set of 18 SNPs were chosen for replication. One SNP (rs5918890) failed assay design and another SNP (rs6524611) had a call rate <95%, leaving 16 SNPs for analysis.

The maternal triads consisted of the mother of a preterm infant and the infant’s maternal grandparents. For analysis of the maternal triads, 98 case families (293 individuals) were eligible for analysis. Of these, 116 individuals were excluded because they had a call rate <90% and 45 individuals were subsequently excluded because there was only one person left in the family. This left 53 families (132 individuals) for further analysis. None of the SNPs had a call rate <95%, but one SNP was excluded because of low MAF. Thus, 9 SNPs were included in the analysis.

#### The argentina prematurity study merged with the US prematurity study and the denmark family study of PTD

To increase statistical power in the maternal replication study, 103 Argentinean families (309 individuals) were added to the analysis, yielding a total of 201 families (602 individuals). After excluding 4 families (12 individuals ) with Mendelian errors, 183 individuals with call rate <90%, 51 individuals with no family left in the analysis and 4 parents with no infant left, only 140 families (352 individuals) remained for analysis. No SNP had a call rate <95% and all 10 SNPs were thus included in the analysis.

### Data Analysis

The Norwegian and Danish case-mother dyads and control-mother dyads were analyzed separately using a hybrid approach in the *R* statistical package Haplin [22], [33], [34]. The software is freely downloadable at http://www.uib.no/smis/gjessing/genetics/software/haplin. We analyzed both maternal and fetal SNP effects. In addition, separate analyses were performed for male and female cases to identify possible sex-specific effects. A Bonferroni correction puts the significance threshold at 4.1**×**10**^−^** ^6^ for this study.

**Figure 1:**
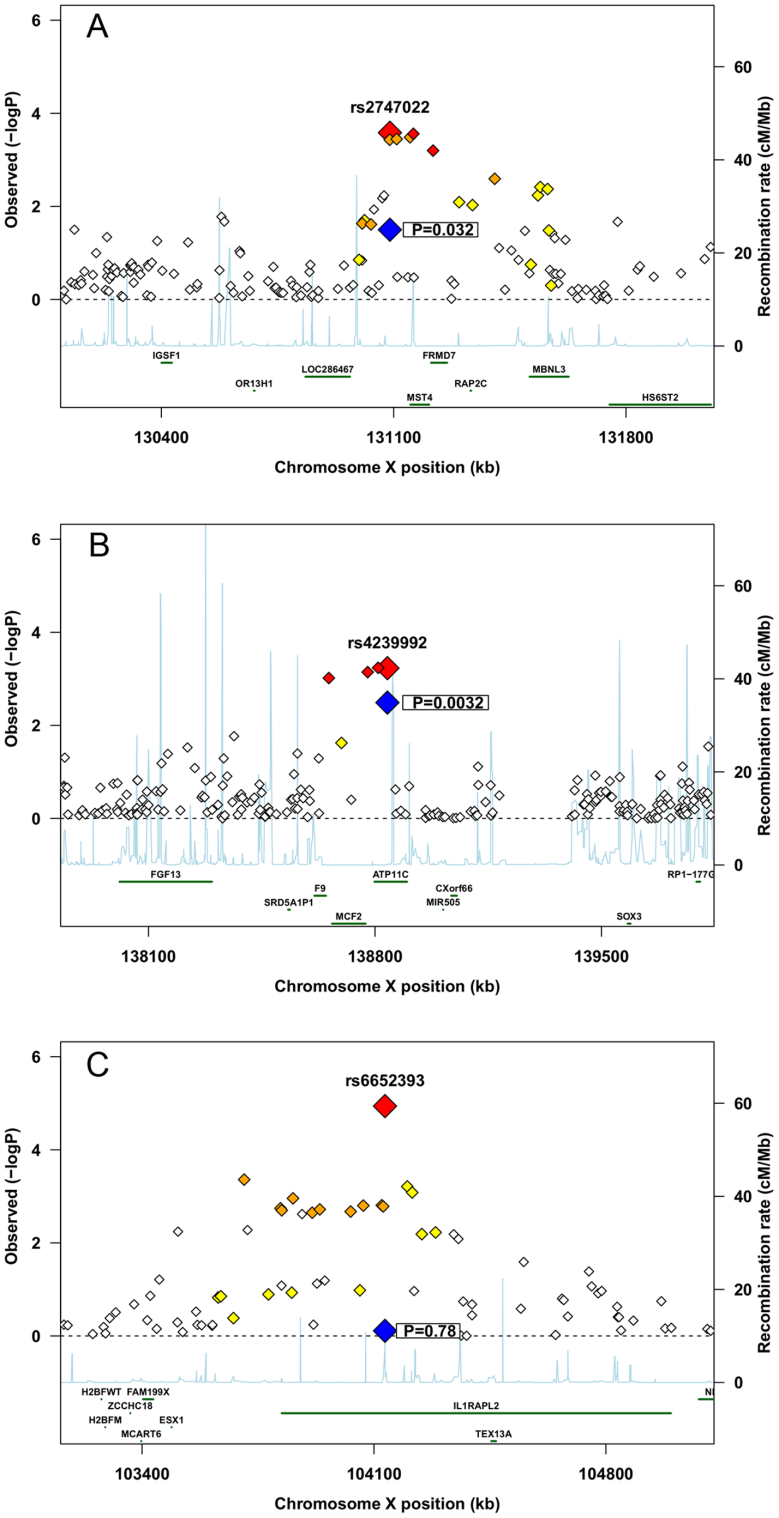
Regional association plots. A) rs2747022 in mothers, B) rs4239992 in infants, C) rs6652393 in male infants. Large red diamond represents the association in the meta-analysis. Large blue diamond represents the association in the replication study. Small red, orange and yellow diamonds represents SNPs in different degrees of LD with the associated SNP.

The results from the two populations were combined in a fixed-effects meta-analysis of 12,211 SNPs in 1,535 case-mother dyads and 1,487 control-mother dyads. Thus, for each SNP, the weighted average of the two log(RR) values was computed using inverse variance weights, and similarly for the standard error of the combined estimate. In addition, the two overall p-values were combined using Fisher’s method [35], and quantile-quantile (QQ) plots were generated for the combined p-values for each of the different analyses (Figure S1). In addition, regional association plots for the SNPs with the lowest uncorrected p-values were made using a modified version of the *R* script available at http://www.broadinstitute.org/files/shared/diabetes/scandinavs/assocplot.R (Figure 1). With the meta-analysis approach, relative risks with opposing directions in the two populations may cancel each other out, whereas the Fisher method combines p-values irrespective of the direction of the effect.

The replication data from the US prematurity study were analyzed using the case-parent triad module in Haplin. In this setting, the unit of analysis is a preterm infant and his/her parents/siblings. The analyzed maternal triads consisted of the mother of a preterm infant and her parents (the infant’s maternal grandparents).

Haplin was originally designed to analyze genetic and environmental risk factors using a case-parent triad approach, a case-control approach or a combination of the two [34]. In addition to fetal effects, Haplin can also estimate maternal effects unambiguously. Haplin is based on log-linear modeling and uses a full maximum likelihood (ML) model for estimation of relative risks. In addition, missing genotypes are imputed using the expectation maximization (EM) algorithm.

The relationship between male and female allele effects may be influenced by X inactivation in females. The analyses were therefore carried out using a parameterization model in which boys and girls were assigned different baseline risks (B_B_ and B_G_) and a shared relative risk (RR). By assuming separate baseline risks, confounding due to other effects that can influence sex differences can be avoided. The baseline risk applies when no risk alleles are present (i.e. A_1_ in boys and A_1_A_1_ in girls, with A_2_ representing the risk allele). The risk increases similarly in boys and girls (B_B_*RR and B_G_*RR) when they are hemizygous for the risk allele (A_2_ in boys) or homozygous for the risk allele (A_2_A_2_ in girls). When girls are heterozygous (A_1_A_2_), the risk is the average of B_G_ and B_G_*RR. X inactivation is thus taken into account. In addition to joint analyses of males and females, Haplin has an option for running sex-specific analyses [22].

## Results

Demographic and pregnancy characteristics for cases and controls in DNBC and MoBa are outlined in Table 1. Table 2 shows the number of families in the replication study.

**Table 1:** Demographic and pregnancy characteristics for the cases and controls in the Danish and Norwegian GWAS.

	DNBC			MoBa		
	Cases (n = 1,046)	Controls (n = 959)	p	Cases (n = 489)	Controls (n = 528)	p
Maternal age (years)	26 (17–44)	30 (18–42)	<0.001	28.7 [20]–[34]	29.4 [20]–[34]	0.003
Gestational age (days)	246 (142–258)	283 (273–293)	<0.001	251.8 [172–256]	280.5 [273–286]	<0.001
Birth weight (g)*	2550 (600–4040)	3700 (2165–5250)	<0.001	2760 [747–3840]	3670 [2610–4950]	<0.001
Male	573 (54.8%)	502 (52.4%)	0.28	265 (54.2%)	247 (46.8%)	0.018
Previous PTD**	62 (19.9%)	20 (3.2%)	<0.001	49 (25.5%)	12 (4,0%)	<0.001
Primiparous***	725 (70.2%)	341 (35.6%)	<0.001	297 (60.7%)	226 (42.8%)	<0.001

DNBC – Danish National Birth Cohort; MoBa – Norwegian Mother and Child Cohort Study.

For continuous variables, median is reported with the range in brackets.

* 16 missing for cases and 4 missing for controls in DNBC.

** Previous PTD based on data for multipara only, 4 missing for cases and 4 missing for controls in DNBC.

*** 13 missing for cases and 1 missing for controls in DNBC.

**Table 2:** Case families in the replication study.

	Maternal US	Maternal Denmark	Maternal Argentina	Fetal
Full triads	22	4	46	182
Mother-infant dyads	23	2	38	66
Father-infant dyads	2	0	3	14
Mother, father, two siblings	0	0	0	4
Mother, father, three siblings	0	0	0	1
Total number of families	47	6	87	267
Total number of individuals	116	16	220	727

### Maternal Effects

Of 12,211 analyzed maternal SNPs, 29 had a combined p-value <10**^−^** ^3^ before Bonferroni correction for multiple testing and 14 of these had an uncorrected p-value <0.05 in both the Norwegian and Danish population (Table S1). The best result was for rs7892483, with a combined p-value of 9.8**×**10**^−^** ^6^ and a relative risk (RR) of 1.7 (95% CI: 1.3, 2.1; Table 3). However, none of the SNPs remained significant after correcting for multiple testing.

**Table 3:** Maternal results from the two independent GWAS and the combined analysis.

			DNBC		MoBa		Combined analysis	
Gene	SNP	Alleles	MAF	RR 95% CI	MAF	RR 95% CI	RR 95% CI	p RR
	rs7892483	A/g	0.03	1.76 (1.29, 2.37)	0.05	1.58 (1.08, 2.30)	1.69 (1.34, 2.13)	9.81E-06
	rs5972070	a/G	0.03	1.65 (1.21, 2.23)	0.05	1.62 (1.10, 2.36)	1.64 (1.30, 2.08)	3.67E-05
	rs6619677*	a/C	0.03	1.59 (1.16, 2.17)	0.03	1.81 (1.17, 2.77)	1.67 (1.30, 2.14)	5.85E-05
*REPS2*	rs12557633	a/G	0.08	1.42 (1.14, 1.75)	0.09	1.34 (1.00, 1.78)	1.39 (1.17, 1.64)	1.54E-04
	rs4562494	a/C	0.28	0.82 (0.71, 0.94)	0.28	0.78 (0.63, 0.96)	0.80 (0.72, 0.90)	2.14E-04
*FRMD7*	rs2747022	A/g	0.31	1.17 (1.02, 1.33)	0.31	1.33 (1.10, 1.61)	1.22 (1.10, 1.36)	2.62E-04
*FGD1*	rs3213533*	A/g	0.15	0.73 (0.60, 0.88)	0.14	0.82 (0.63, 1.07)	0.76 (0.65, 0.88)	3.40E-04
*IL1RAPL1*	rs5927786**	a/G	0.47	0.79 (0.69, 0.89)	0.48	0.94 (0.78, 1.13)	0.84 (0.75, 0.93)	5.77E-04
*IL1RAPL1*	rs4829104	A/g***	0.52	1.25 (1.42, 1.10)	0.49	1.08 (0.90, 1.28)	1.19 (1.07, 1.32)	8.39E-04
*COL4A6*	rs2295912	a/G	0.02	0.50 (0.30, 0.84)	0.02	048 (0.22, 1,04)	0.50 (0.33, 0.76)	1.30E-03

* deviates from HWE in the Norwegian population; failed replication analysis in Haplin.

** deviates from HWE in the Danish population.

*** a/G in the Danish population.

Abbreviations: MAF, minor allele frequency; RR, relative risk; 95% CI, 95% confidence interval; HWE, Hardy Weinberg Equilibrium.

### Fetal Effects

Of the analyzed fetal SNPs, 19 had a combined p-value <10**^−^** ^3^ before Bonferroni correction and 9 of these had an uncorrected p-value <0.05 in both populations in the discovery phase (Table S2). None of the SNPs reached the chromosome-wide significance level threshold of 4.1**×**10**^−^** ^6^, but rs2961403 was closest to significance, with a combined p-value of 1.2**×**10**^−^** ^5^ and a RR of 1.3 (95% CI: 1.1, 1.4) (Table 4). However, this SNP was only borderline significant in the Norwegian sample (p-value = 1.8**×**10**^−^** ^6^). In the Danish study, rs6528251 in the “DEAD/H (Asp-Glu-Ala-Asp/His) box polypeptide 26B” (*DDX26B*) gene was the SNP closest to significance (p-value = 1.1**×**10**^−^** ^4^).

**Table 4:** Fetal results from the two independent GWAS and the combined analysis.

			DNBC		MoBa		Combined analysis		
Gene	SNP	Alleles	MAF	RR (95% CI)	MAF	RR (95% CI)	RR (95% CI)	p RR	p overall
	rs2961403	a/G	0.07	1.06 (0.91, 1.23)	0.06	1.63 (1.34, 1.97)	1.25 (1.11, 1.40)	1.50E-04	1.19E-05
	rs3008952	a/G	0.07	1.06 (0.91, 1.23)	0.06	1.63 (1.35, 1.97)	1.25 (1.11, 1.40)	1.57E-04	1.22E-05
*UTP14A*	rs2273021	A/g	0.10	0.98 (0.85, 1.12)	0.09	1.48 (1.25, 1.74)	1.16 (1.04, 1.29)	5.70E-03	1.25E-04
*PLAC1*	rs12557773	A/g	0.40	1.13 (1.04, 1.22)	0.46	0.84 (0.75, 0.95)	1.03 (0.96, 1.10)	4.20E-01	3.70E-04
	rs5919596	A/g	0.11	0.85 (0.74, 0.98)	0.11	0.70 (0.55, 0.88)	0.81 (0.72, 0.91)	3.80E-04	3.88E-04
	rs2961408	a/C	0.11	1.09 (0.96, 1.23)	0.10	1.40 (1.18, 1.66)	1.19 (1.07, 1.31)	7.94E-04	4.40E-04
	rs3008935	A/g	0.11	1.14 (1.01, 1.29)	0.11	1.33 (1.12, 1.56)	1.20 (1.09, 1.32)	1.74E-04	5.44E-04
	rs2485729	a/G	0.02	0.54 (0.35, 0.84)	0.03	1.40 (1.05, 1.86)	1.06 (0.84, 1.33)	6.52E-01	5.79E-04
*MIR505*	rs4239992	A/g	0.02	0.55 (0.35, 0.85)	0.03	1.42 (1.07, 1.88)	1.08 (0.85, 1.36)	5.28E-01	5.90E-04
	rs5918890	a/G	0.11	0.88 (0.76, 1.00)	0.11	0.70 (0.55, 0.87)	0.82 (0.73, 0.92)	1.02E-03	6.39E-04
	rs714073	a/G	0.23	1.12 (1.02, 1.23)	0.22	1.23 (1.07, 1.40)	1.15 (1.07, 1.24)	1.38E-04	6.50E-04
*IL1RAPL2*	rs6652393	a/G	0.40	1.07 (0.99, 1.16)	0.41	1.23 (1.09, 1.39)	1.12 (1.05, 1.20)	7.18E-04	7.05E-04
	rs5953790	a/C	0.02	0.56 (0.36, 0.85)	0.03	1.42 (1.06, 1.88)	1.07 (0.85, 1.35)	5.73E-01	7.17E-04
*TLR7*	rs5743749	a/G	0.08	0.77 (0.64, 0.91)	0.08	0.78 (0.61, 1.00)	0.77 (0.67, 0.89)	2.59E-04	7.43E-04
	rs714075	A/g	0.30	1.06 (0.97, 1.15)	0.29	1.26 (1.11, 1.42)	1.12 (1.04, 1.20)	1.57E-03	7.65E-04
*ATP11C*	rs17328647	A/g	0.02	0.55 (0.35, 0.85)	0.03	1.38 (1.03, 1.81)	1.05 (0.84, 1.33)	6.59E-01	9.62E-04
*AMOT*	rs632783	A/c	0.15	1.20 (1.08, 1.34)	0.15	1.11 (0.95, 1.30)	1.18 (1.08, 1.28)	2.13E-04	1.08E-03
*DACH2*	rs6524611	a/G	0.40	1.08 (1.00, 1.18)	0.39	1.21 (1.07, 1.36)	1.12 (1.05, 1.20)	6.98E-04	1.24E-03

### Sex-stratified Analyses

When girls and boys were analyzed separately, 10 SNPs had a combined, uncorrected p-value <10**^−^** ^3^ for boys and five of these SNPs had a p-value <0.05 in both studies (Table S3). The SNP closest to significance was rs6652393 in the interleukin-1 receptor-associated protein-like 2 gene (*IL1RAPL2*) (Table 5). In boys, the A-allele of this SNP had an overall p-value of 3.1**×**10**^−^** ^5^ and a RR of 1.2 (95% CI: 1.1, 1.3). Moreover, three of the other top SNPs were located in *IL1RAPL2* (Table S3). For girls, there were 17 SNPs with a combined p-value <10**^−^** ^3^, only one of which had a p-value <0.05 in both studies (Table 6, Table S4).

**Table 5:** Sex-stratified analysis, males.

			DNBC		MoBa		Combined analysis		
Gene	SNP	Alleles	MAF	RR (95% CI)	MAF	RR (95% CI)	RR (95% CI)	p RR	p overall
*IL1RAPL2*	rs6652393	a/G	0.38	1.14 (1.04, 1.26)	0.40	1.32 (1.15, 1.52)	1.20 (1.11, 1.29)	5.47E-06	1.15E-05
	rs3008952	a/G	0.07	1.10 (0.92, 1.30)	0.05	1.68 (1.33, 2.12)	1.28 (1.11, 1.46)	4.60E-04	7.50E-05
	rs2961403	a/G	0.07	1.10 (0.92, 1.30)	0.06	1.66 (1.31, 2.10)	1.27 (1.11, 1.46)	5.46E-04	1.03E-04
	rs3131391	A/g*	0.31	1.18 (1.07, 1.31)	0.29	1.20 (1.03, 1.39)	1.19 (1.10, 1.29)	3.16E-05	2.23E-04
*IL1RAPL2*	rs5962953	A/g	0.42	1.11 (1.01, 1.22)	0.44	1.27 (1.10, 1.45)	1.16 (1.07, 1.25)	2.53E-04	4.37E-04

The five most significant SNPs.

**Table 6:** Sex-stratified analysis, females.

			DNBC		MoBa		Combined analysis		
Gene	SNP	Alleles	MAF	RR (95% CI)	MAF	RR (95% CI)	RR (95% CI)	p RR	p overall
*DACH2*	rs761202	A/g	0.20	0.89 (0.73, 1.09)	0.20	1.80 (1.39, 2.32)	1.17 (1.00, 1.36)	4.96E-02	3.05E-05
*DACH2*	rs724620	A/g	0.21	0.88 (0.72, 1.07)	0.20	1.78 (1.37, 2.28)	1.15 (0.99, 1.35)	6.70E-02	3.28E-05
*GRIA3*	rs5958198	a/C	0.33	1.45 (1.23, 1.71)	0.37	1.06 (0.84, 1.33)	1.30 (1.14, 1.49)	9.01E-05	8.77E-05
*GRIA3*	rs6608062	A/g	0.33	1.44 (1.22, 1.69)	0.37	1.01 (0.80, 1.27)	1.28 (1.12, 1.46)	2.40E-04	1.79E-04
*H2BFM*	rs1024325	A/g	0.35	0.70 (0.58, 0.83)	0.35	0.97 (0.77, 1.23)	0.78 (0.68, 0.90)	5.14E-04	3.65E-04

The five most significant SNPs.

### Replication Analyses

In the maternal replication study, SNP rs2747022 located in *FRMD7* had an uncorrected p-value of 0.01 (Table 7). After including the Argentinean families, this SNP had an uncorrected p-value of 0.03 (Table 8, Figure 1A). In the fetal replication, four SNPs had p-values <0.05: rs4239992 in *MIR-505* (microRNA 505), rs17328647 in *ATP11C* (ATPase, class 6 type, 11C), and rs5953790 and rs2485729 located close to the *ATP11C* gene region (Table 9, Figure 1B). All of the SNPs were in strong linkage disequilibrium (LD) with each other and the G allele at rs4239992 increased the relative risk of spontaneous PTD by 2.3 (95% CI: 1.3, 4.1). In the sex-stratified analysis, there was suggestive evidence of association with these SNPs in boys, but not in girls.

**Table 7:** Replication of maternal SNPs in the US and Denmark study.

Gene	SNP	Alleles	MAF	RR (95% CI)	p RR	p overall
*FRMD7*	rs2747022	A/g	0.23	2.59 (1.18, 5.57)	0.018	0.014
*IL1RAPL1*	rs5927786	a/G	0.32	1.83 (0.88, 3.74)	0.105	0.097
*FGD1*	rs3213533	A/g	0.06	2.18 (0.58, 7.82)	0.251	0.216
	rs6619677	a/C	0.04	0.23 (0.02, 2.49)	0.232	0.221
*IL1RAPL1*	rs4829104	a/G	0.33	1.42 (0.68, 2.91)	0.346	0.343
	rs4562494*	a/C	0.29	0.72 (0.32, 1.55)	0.394	0.410
*REPS2*	rs12557633	a/G	0.04	1.63 (0.32, 7.88)	0.555	0.539
	rs7892483	A/g	0.06	0.57 (0.09, 3.37)	0.538	0.549
	rs5972070	a/G	0.04	0.70 (0.11, 4.20)	0.703	0.704

* Deviates from HWE.

**Table 8:** Replication of maternal SNPs in the US, Denmark and Argentina studies.

Gene	SNP	Alleles	MAF	RR (95% CI)	p RR	p overall
*FRMD7*	rs2747022	A/g	0.16	1.80 (1.03, 3.08)	0.040	0.032
	rs7892483	A/g	0.04	0.43 (0.12, 1.48)	0.181	0.193
	rs4562494	a/C	0.46	0.84 (0.54, 1.27)	0.402	0.416
	rs6619677	a/C	0.10	0.74 (0.36, 1.51)	0.409	0.424
*FGD1*	rs3213533	A/g	0.24	1.14 (0.69, 1.85)	0.614	0.622
*REPS2*	rs12557633	a/G	0.07	1.19 (0.53, 2.61)	0.671	0.670
*IL1RAPL1*	rs5927786	A/g	0.43	1.10 (0.71, 1.69)	0.674	0.684
*IL1RAPL1*	rs4829104	A/g	0.45	1.09 (0.71, 1.66)	0.683	0.691
	rs5972070*	a/G	0.04	0.85 (0.27, 2.52)	0.766	0.766
*COL4A6*	rs2295912	a/G	0.04	1.04 (0.38, 2.74)	0.940	0.946

**Table 9:** Replication of fetal SNPs in the US study.

			Overall				Males	Females
	snp	alleles	MAF	RR (95% CI)	p RR	p overall	RR (95% CI)	RR (95% CI)
*MIR505*	rs4239992	A/g	0.01	2.30 (1.28, 4.06)	0.005	0.003	2.85 (1.29, 6.02)	1.42 (0.28, 7.00)
	rs5953790	a/C	0.01	2.36 (1.30, 4.17)	0.005	0.003	2.30 (1.17, 4.45)	2.38 (0.27, 19.67)
	rs2485729	a/G	0.01	2.09 (1.20, 3.55)	0.008	0.008	2.31 (1.18, 4.47)	1.41 (0.27, 6.85)
*ATP11C*	rs17328647	A/g	0.01	2.08 (1.19, 3.60)	0.010	0.008	2.32 (1.18, 4.38)	1.44 (0.28, 7.06)
*AMOT*	rs632783	A/c	0.13	1.24 (0.96, 1.58)	0.104	0.102	1.29 (0.98, 1.69)	0.99 (0.51, 1.90)
	rs5919596	A/g	0.07	1.24 (0.90, 1.68)	0.184	0.190	1.20 (0.83, 1.73)	1.81 (0.75, 4.24)
	rs714075	A/g	0.30	0.88 (0.72, 1.08)	0.205	0.204	0.86 (0.69, 1.07)	1.01 (0.59, 1.68)
	rs714073	a/G	0.23	0.92 (0.73, 1.14)	0.413	0.423	0.89 (0.70, 1.14)	1.03 (0.59, 1.79)
	rs12557773	A/g	0.41	1.08 (0.89, 1.29)	0.439	0.442	1.14 (0.93, 1.38)	0.74 (0.46, 1.20)
	rs3008952	a/G	0.08	1.10 (0.79, 1.51)	0.572	0.577	1.08 (0.75, 1.56)	1.26 (0.53, 2.89)
	rs2961403	a/G	0.08	1.10 (0.79, 1.51)	0.579	0.584	1.08 (0.75, 1.56)	1.24 (0.53, 2.86)
	rs3008935	A/g	0.13	0.96 (0.72, 1.26)	0.766	0.760	0.87 (0.63, 1.19)	1.59 (0.75, 3.33)
*UTP14A*	rs2273021	A/g	0.11	0.97 (0.72, 1.29)	0.831	0.830	0.90 (0.65, 1.22)	1.68 (0.69, 3.96)
	rs6652393	a/G	0.40	1.00 (0.84, 1.21)	0.966	0.953	1.03 (0.84, 1.25)	0.82 (0.50, 1.34)
*TLR7*	rs5743749	a/G	0.09	1.01 (0.74, 1.36)	0.967	0.979	1.03 (0.72, 1.46)	0.91 (0.42, 1.93)
	rs2961408	a/C	0.11	1.00 (0.75, 1.33)	0.970	0.983	0.93 (0.66, 1.30)	1.52 (0.71, 3.18)

## Discussion

In this meta-analysis of two independent GWAS from two ancestrally and geographically closely related populations, many SNPs had p-values <0.05 but none remained significant after a Bonferroni adjustment for multiple testing. Replication was attempted for the SNPs closest to significance, but the results were inconclusive.

### Maternal Effects

For the maternal SNPs, the most promising finding was rs2747022 in *FRMD7*. Before Bonferroni correction, the G allele at this SNP was associated with an increased risk of spontaneous PTD in the Norwegian and Danish data, the combined analysis and the replication study, also when the Argentinean families were included. Another SNP in this gene, rs7880476, was also associated in the combined analyses of the Norwegian and Danish data. But like rs2747022 above, the association was no longer significant after Bonferroni correction.

*FRMD7* maps to Xq26.2 and is associated with X-linked idiopathic congenital nystagmus [36]. This condition is fully penetrant in males, but has incomplete penetrance in females [36]. Expression analyses show that mRNA is present at low levels in most human adult tissues. In embryos, there is expression in various parts of the brain [36], and it has been postulated that the FRMD7 protein is important for neurite development and neuronal differentiation [37]. It is unclear how this gene might be involved in the pathogenesis of spontaneous PTD. An associated SNP may be a surrogate for etiologic variants adjacent to the gene in which the most associated SNP is located. However, the genes immediately flanking *FRMD7* (*MST4, RAP2C, MBNL3*) do not appear to be obvious candidates for PTD, although *MBNL3* is involved in alternative splicing, a mechanism that would be consistent with altered developmental expression patterns.

The SNP closest to significance in the combined analysis was rs7892483. This finding was consistent across the Norwegian and Danish samples, but not in the replication study involving the US and Argentinean samples. Of the other most associated SNPs, four (rs5972070, rs5972071, rs5973734, and rs5973741; Table S1) were in strong LD with rs7892483. These SNPs lie in a gene desert and have not previously been associated with any disease. It is possible that they are in LD with a causal variant of unknown location. Several loci associated with disease have been localized to gene deserts, and it has been shown that these regions may harbor regulatory elements that can modulate gene expression over large distances on the chromosome [38].

### Fetal Effects

Before Bonferroni correction, the closely linked SNPs rs4239992, rs17328647, r5953790 and rs2485729s in the MIR505/ATPC11 gene region had uncorrected p-values <0.05, both in the combined analysis and in the replication study. However, the relative risks in the Norwegian and Danish samples point in opposite directions, complicating the interpretation of these risk estimates. This could be explained by a genetic “flip-flop”, a phenomenon characterized by opposite alleles being associated in two populations owing to heterogeneous effects of the same variant or to differences in LD [39]. However, this is unlikely given the similar allele frequencies of these SNPs in the Norwegian and Danish samples and the close ancestral origins of these two populations. Nevertheless, this is an interesting finding, particularly because rs4239992 is located in a microRNA gene. These genes code for small RNA molecules that can have important regulatory functions in gene expression [40], and might thus be involved in regulating genes important for PTD.

The six top hits in the combined analysis were also among the top hits in the Norwegian but not the Danish study. Since analyses based on the hybrid study design are not fully protected against population stratification because of the case-control component, some of the hits might be the result of population substructure or random false positives. However, none of these SNPs achieved chromosome-wide significance in the replication study.

### Sex-stratified Analyses

In the combined study, the most promising SNP among males was rs6652393 in *IL1RAPL2.* Before Bonferroni correction this SNP was significant at p<0.05 in both populations, but not in the sex-stratified analysis of females, indicating a possible sex-specific effect. *IL1RAPL2* maps to Xq22 and is a member of the IL-1 receptor family. In mice, *IL1RAPL2* is specifically expressed in the central nervous system (CNS) from embryonic day 12.5 onwards [41]. A study by Born and co-workers [42] detected expression of *IL1RAPL2* in skin, liver, placenta, and fetal brain tissues. Only weak expression has been detected in the adult brain [41]. *IL1RAPL2* is closely related to *IL1RAPL1,* which has been associated with mental retardation, autism and psychiatric disorders [43], all of which are conditions associated with PTD [2]. Mechanisms leading to PTD may also be responsible for impaired neonatal outcome [44], with boys being more vulnerable to these outcomes than girls. Therefore, it is plausible that some sex-specific mechanism involved in both CNS development and spontaneous PTD is at play. As mentioned earlier, there is a higher level of the IL-1 receptor antagonist in the amniotic fluid of females compared with males. It has also been shown that IL-1 can induce PTD in mice [41], but it remains unclear how *IL1RAPL2* is involved in the response to IL-1 [42].

In the sex-stratified analysis, there was only suggestive evidence of association with one SNP across both study samples in females. However, the respective relative risks were in opposite directions in the Norwegian and Danish samples. In the Norwegian sample, several SNPs in the dachshund homolog 2 gene (*DACH2)* were close to significance in females, whereas in the Danish study the SNPs closest to significance were in the glutamate receptor 3 gene (*GRIA3)*. These findings were not replicated and are likely to be false positives. Further, none of the associations remained after correction for multiple testing. Taken together, our data do not provide strong evidence for a sex-specific effect in girls in the combined analysis.

In the sex-stratified replication, the same SNPs that were promising in the fetal replication study were also promising in males but not females, again indicating a possible sex-specific effect. However, this effect was not present in the original study population. Again, none of the replicated SNPs were significant in females.

Despite the negative results, this is the first study to look for associations between spontaneous PTD and SNPs along the X chromosome. The hybrid design used in this study is widely applicable to other perinatal disorders and has several attractive and novel features compared with other methods. It is less prone to population stratification than the case-control design, and since it involves more controls than the case-parent triad design alone, it not only provides more statistical power for detecting an effect, it also allows the main effects of an exposure (genetic or environmental) to be estimated. Furthermore, our study subjects come from two relatively homogeneous populations that share a common ancestry and geography, further protecting against population stratification. Because spontaneous PTD is a heterogeneous condition, we applied strict inclusion criteria in order to obtain a clearly defined phenotype.

Although this study is based on one of the largest collections of PTD samples to date, the power to detect small genetic effects in particular may still be limited. Several associations that were significant in the two independent discovery populations from Denmark and Norway failed to replicate in a third study population from the US. There are several plausible explanations for this. First of all, the associations did not remain significant after a Bonferroni correction for multiple testing, increasing the likelihood that they were false positives. Whereas the primary studies were based on a relatively homogeneous Scandinavian population, the replication study was based on a more heterogeneous/admixed US and Argentinean population. Also, some of the variables that were used as exclusion criteria in the primary studies (for example diabetes and cerclage) were not available for the replication population. Furthermore, the replication study had a smaller sample size than the combined study, in particular for the evaluation of maternal SNPs. Finally, the case-parent triad study design in the replication study offers better protection against population stratification than the hybrid design in the primary study, but the statistical power in the former is lower because fewer control alleles are available for comparison.

In conclusion, our data did not provide any strong evidence for the involvement of X-chromosomal SNPs in the risk of spontaneous PTD. However, there were several interesting findings, such as the maternal SNP rs2747022 in *FRMD7*, which represents a particularly attractive candidate for further investigations in other large studies. The hybrid study design described here and the analytic opportunities provided by Haplin should prove valuable not only for the replication but also for exploring other perinatal disorders.

## Supporting Information

Figure S1 **QQ-plots for the overall p-values in the meta-analysis, p-values are combined using Fisher’s method.** A) Maternal SNPs, B) Fetal SNPs, C) Male fetal SNPs, D) Female fetal SNPs.
(TIF)

Table S1 **Maternal results for SNPs with p<1.00×10^−3^.**
(DOCX)

Table S2 **Fetal results for SNPs with p<1.00×10^−3^.**
(DOCX)

Table S3 **Sex-stratified analysis, males, p<1.00×10^−3^.**
(DOCX)

Table S4 **Sex-stratified analysis, females, p<1.00×10^−3^.**
(DOCX)
