# Supplementary figures and images for: X-Chromosomal Maternal and Fetal SNPs and the Risk of Spontaneous Preterm Delivery in a Danish/Norwegian Genome-Wide Association Study
Source: PLoS One. 2013 Apr 16;8(4):e61781. doi: 10.1371/journal.pone.0061781 (PMC3628886; doi:10.1371/journal.pone.0061781)

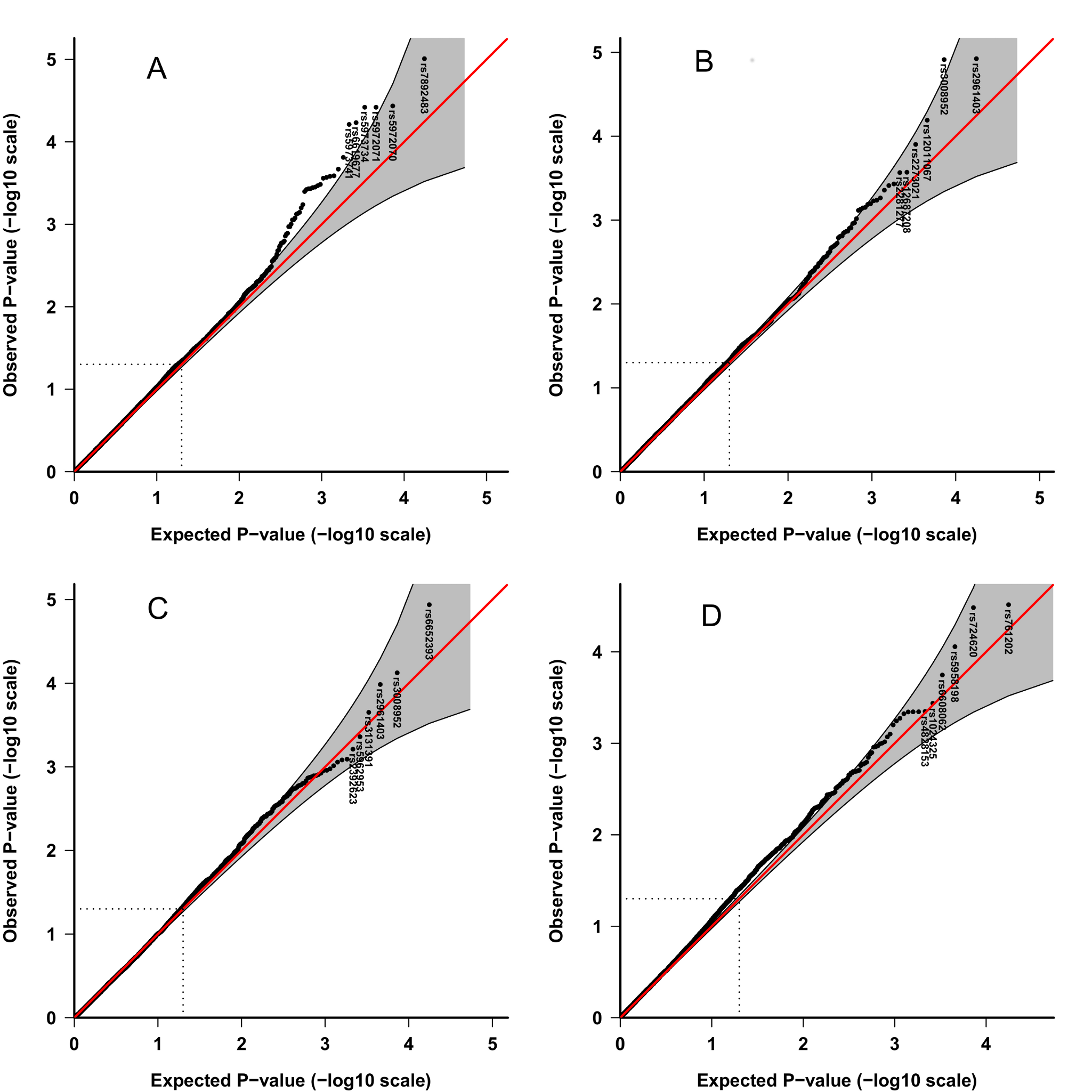

Supplement: Figure S1 — QQ-plots for the overall p-values in the meta-analysis, p-values are combined using Fisher’s method. A) Maternal SNPs, B) Fetal SNPs, C) Male fetal SNPs, D) Female fetal SNPs. (TIF) [file pone.0061781.s001.tif]
