# Supplementary material for: X-Chromosomal Maternal and Fetal SNPs and the Risk of Spontaneous Preterm Delivery in a Danish/Norwegian Genome-Wide Association Study
Source: PLoS One. 2013 Apr 16;8(4):e61781. doi: 10.1371/journal.pone.0061781 (PMC3628886; doi:10.1371/journal.pone.0061781)
Supplement: Table S1 — Maternal results for SNPs with p<1.00×10^−3^. (DOCX) [file pone.0061781.s002.docx]

**Supplemental Table 1. Maternal results for SNPs with p<1.00x10^-3^**

|  |  |  | MoBa | | DNBC | | Combined analysis | |
| --- | --- | --- | --- | --- | --- | --- | --- | --- |
| Gene | SNP | Alleles | MAF | RR | MAF | RR (95% CI) | RR (95% CI) | P RR |
|  | rs7892483 | A/g | 0.05 | 1.58 (1.08, 2.30) | 0.03 | 1.76 (1.29, 2.37) | 1.69 (1.34, 2.13) | 9.81E-06 |
|  | rs5972070 | a/G | 0.05 | 1.62 (1.10, 2.36) | 0.03 | 1.65 (1.21, 2.23) | 1.64 (1.30, 2.08) | 3.67E-05 |
|  | rs5972071 | a/C | 0.05 | 1.62 (1.10, 2.36) | 0.03 | 1.65 (1.21, 2.23) | 1.64 (1.30, 2.07) | 3.80E-05 |
|  | rs5973734 | a/G | 0.05 | 1.62 (1.10, 2.36) | 0.03 | 1.65 (1.21, 2.23) | 1.64 (1.30, 2.07) | 3.80E-05 |
|  | rs6619677* | a/C | 0.03 | 1.81 (1.17, 2.77) | 0.03 | 1.59 (1.16, 2.17) | 1.67 (1.30, 2.14) | 5.85E-05 |
|  | rs5973741 | A/g | 0.05 | 1.60 (1.08, 2.33) | 0.03 | 1.62 (1.19, 2.18) | 1.61 (1.28, 2.03) | 6.14E-05 |
| REPS2 | rs12557633 | a/G | 0.09 | 1.34 (1.00, 1.78) | 0.08 | 1.42 (1.14, 1.75) | 1.39 (1.17, 1.64) | 1.54E-04 |
|  | rs4562494 | a/C | 0.28 | 0.78 (0.63, 0.96) | 0.28 | 0.82 (0.71, 0.94) | 0.80 (0.72, 0.90) | 2.14E-04 |
|  | rs5928689 | a/G | 0.21 | 0.74 (0.59, 0.93) | 0.22 | 0.81 (0.69, 0.95) | 0.79 (0.69, 0.90) | 2.58E-04 |
| FRMD7 | rs2747022 | A/g | 0.31 | 1.33 (1.10, 1.61) | 0.31 | 1.17 (1.02, 1.33) | 1.22 (1.10, 1.36) | 2.62E-04 |
|  | rs4474171 | a/G | 0.28 | 0.79 (0.64, 0.96) | 0.28 | 0.82 (0.71, 0.94) | 0.81 (0.72, 0.91) | 2.70E-04 |
| RAP2C | rs5933120 | A/g | 0.31 | 1.33 (1.10, 1.61) | 0.31 | 1.17 (1.02, 1.33) | 1.22 (1.10, 1.36) | 2.76E-04 |
|  | rs5933118 | A/g | 0.25 | 1.16 (0.95, 1.42) | 0.25 | 1.26 (1.09, 1.45) | 1.23 (1.10, 1.38) | 3.28E-04 |
| FGD1 | rs3213533* | A/g | 0.14 | 0.82 (0.63, 1.07) | 0.15 | 0.73 (0.60, 0.88) | 0.76 (0.65, 0.88) | 3.40E-04 |
|  | rs5914143 | a/G | 0.14 | 0.82 (0.63, 1.07) | 0.15 | 0.73 (0.60, 0.88) | 0.76 (0.65, 0.88) | 3.54E-04 |
|  | rs5933092 | a/C | 0.25 | 1.17 (0.95, 1.43) | 0.25 | 1.26 (1.09, 1.45) | 1.23 (1.10, 1.38) | 3.59E-04 |
| GNL3L | rs5915139 | A/g | 0.14 | 0.82 (0.63, 1.07) | 0.15 | 0.73 (0.60, 0.88) | 0.76 (0.65, 0.88) | 3.69E-04 |
| FRMD7 | rs7880476 | A/g | 0.25 | 1.16 (0.95, 1.42) | 0.25 | 1.26 (1.09, 1.45) | 1.23 (1.10, 1.38) | 3.71E-04 |
| GNL3L | rs6612165 | A/g | 0.14 | 0.82 (0.63, 1.08) | 0.15 | 0.73 (0.60, 0.88) | 0.76 (0.65, 0.88) | 3.78E-04 |
| REPS2 | rs12557656 | A/g | 0.09 | 1.37 (1.02, 1.84) | 0.08 | 1.37 (1.09, 1.70) | 1.37 (1.15, 1.63) | 4.01E-04 |
| IL1RAPL1 | rs5927786** | a/G | 0.48 | 0.94 (0.78, 1.13) | 0.47 | 0.79 (0.69, 0.89) | 0.84 (0.75, 0.93) | 5.77E-04 |
|  | rs1005488 | a/G | 0.29 | 1.32 (1.09, 1.60) | 0.30 | 1.15 (1.01, 1.32) | 1.21 (1.08, 1.35) | 6.32E-04 |
|  | rs6632141 | a/G | 0.34 | 0.93 (0.77, 1.13) | 0.35 | 0.78 (0.68, 0.89) | 0.83 (0.74, 0.92) | 7.17E-04 |
|  | rs2144678 | A/g | 0.43 | 1.10 (0.92, 1.32) | 0.42 | 1.24 (1.09, 1.40) | 1.19 (1.08, 1.32) | 7.38E-04 |
| FGD1 | rs2268428* | a/G | 0.14 | 0.81 (0.62, 1.06) | 0.14 | 0.75 (0.61, 0.90) | 0.77 (0.66, 0.89) | 7.53E-04 |
| IL1RAPL1 | rs4829104 | A/g*** | 0.49 | 1.08 (0.90, 1.28) | 0.52 | 1.25 (1.42, 1.10) | 1.19 (1.07, 1.32) | 8.39E-04 |
|  | rs7885649 | A/g | 0.34 | 1.09 (0.90, 1.31) | 0.34 | 1.25 (1.09, 1.42) | 1.20 (1.08, 1.33) | 8.81E-04 |
|  | rs4829627 | A/g | 0.15 | 1.13 (0.88, 1.44) | 0.18 | 1.30 (1.10, 1.51) | 1.25 (1.09, 1.42) | 8.84E-04 |
|  | rs5949639* | A/g | 0.40 | 0.90 (0.74, 1.08) | 0.41 | 0.81 (0.71, 0.92) | 0.84 (0.76, 0.93) | 9.95E-04 |

*deviates from HWE in Norwegian sample

**deviates from HWE in Danish sample

***a/G in Danish sample
