# Supplementary material for: X-Chromosomal Maternal and Fetal SNPs and the Risk of Spontaneous Preterm Delivery in a Danish/Norwegian Genome-Wide Association Study
Source: PLoS One. 2013 Apr 16;8(4):e61781. doi: 10.1371/journal.pone.0061781 (PMC3628886; doi:10.1371/journal.pone.0061781)
Supplement: Table S2 — Fetal results for SNPs with p<1.00×10^−3^. (DOCX) [file pone.0061781.s003.docx]

**Supplemental Table 2. Fetal results for SNPs with p<1.00x10^-3^**

|  |  |  | MoBa | | DNBC | | Combined analysis | | |
| --- | --- | --- | --- | --- | --- | --- | --- | --- | --- |
| Gene | SNP | Alleles | MAF | RR (95% CI) | MAF | RR (95% CI) | RR (95% CI) | p RR | p overall |
|  | rs2961403 | a/G | 0.06 | 1.63 (1.34, 1.97) | 0.07 | 1.06 (0.91, 1.23) | 1.25 (1.11, 1.40) | 1.50E-04 | 1.19E-05 |
|  | rs3008952 | a/G | 0.06 | 1.63 (1.35, 1.97) | 0.07 | 1.06 (0.91, 1.23) | 1.25 (1.11, 1.40) | 1.57E-04 | 1.22E-05 |
| UTP14A | rs12011067 | A/g | 0.09 | 1.49 (1.26, 1.75) | 0.10 | 0.97 (0.85, 1.11) | 1.15 (1.04, 1.28) | 6.41E-03 | 6.44E-05 |
| UTP14A | rs2273021 | A/g | 0.09 | 1.48 (1.25, 1.74) | 0.10 | 0.98 (0.85, 1.12) | 1.16 (1.04, 1.29) | 5.70E-03 | 1.25E-04 |
|  | rs12687208 | A/g | 0.09 | 1.45 (1.23, 1.71) | 0.10 | 0.98 (0.85, 1.12) | 1.15 (1.04, 1.28) | 8.34E-03 | 2.69E-04 |
| UTP14A | rs2281277 | A/c | 0.09 | 1.45 (1.22, 1.71) | 0.09 | 0.97 (0.84, 1.12) | 1.15 (1.03, 1.28) | 1.12E-02 | 2.72E-04 |
| PLAC1 | rs12557773 | A/g | 0.46 | 0.84 (0.75, 0.95) | 0.40 | 1.13 (1.04, 1.22) | 1.03 (0.96, 1.10) | 4.20E-01 | 3.70E-04 |
|  | rs5919596 | A/g | 0.11 | 0.70 (0.55, 0.88) | 0.11 | 0.85 (0.74, 0.98) | 0.81 (0.72, 0.91) | 3.80E-04 | 3.88E-04 |
|  | rs2961408 | a/C | 0.10 | 1.40 (1.18, 1.66) | 0.11 | 1.09 (0.96, 1.23) | 1.19 (1.07, 1.31) | 7.94E-04 | 4.40E-04 |
|  | rs3008935 | A/g | 0.11 | 1.33 (1.12, 1.56) | 0.11 | 1.14 (1.01, 1.29) | 1.20 (1.09, 1.32) | 1.74E-04 | 5.44E-04 |
|  | rs2485729 | a/G | 0.03 | 1.40 (1.05, 1.86) | 0.02 | 0.54 (0.35, 0.84) | 1.06 (0.84, 1.33) | 6.52E-01 | 5.79E-04 |
|  | rs4239992 | A/g | 0.03 | 1.42 (1.07, 1.88) | 0.02 | 0.55 (0.35, 0.85) | 1.08 (0.85, 1.36) | 5.28E-01 | 5.90E-04 |
|  | rs5918890 | a/G | 0.11 | 0.70 (0.55, 0.87) | 0.11 | 0.88 (0.76, 1.00) | 0.82 (0.73, 0.92) | 1.02E-03 | 6.39E-04 |
|  | rs714073 | a/G | 0.22 | 1.23 (1.07, 1.40) | 0.23 | 1.12 (1.02, 1.23) | 1.15 (1.07, 1.24) | 1.38E-04 | 6.50E-04 |
| IL1RAPL2 | rs6652393 | a/G | 0.41 | 1.23 (1.09, 1.39) | 0.40 | 1.07 (0.99, 1.16) | 1.12 (1.05, 1.20) | 7.18E-04 | 7.05E-04 |
|  | rs5953790 | a/C | 0.03 | 1.42 (1.06, 1.88) | 0.02 | 0.56 (0.36, 0.85) | 1.07 (0.85, 1.35) | 5.73E-01 | 7.17E-04 |
| TLR7 | rs5743749 | a/G | 0.08 | 0.78 (0.61, 1.00) | 0.08 | 0.77 (0.64, 0.91) | 0.77 (0.67, 0.89) | 2.59E-04 | 7.43E-04 |
|  | rs714075 | A/g | 0.29 | 1.26 (1.11, 1.42) | 0.30 | 1.06 (0.97, 1.15) | 1.12 (1.04, 1.20) | 1.57E-03 | 7.65E-04 |
| ATP11C | rs17328647 | A/g | 0.03 | 1.38 (1.03, 1.81) | 0.02 | 0.55 (0.35, 0.85) | 1.05 (0.84, 1.33) | 6.59E-01 | 9.62E-04 |
