# Supplementary material for: X-Chromosomal Maternal and Fetal SNPs and the Risk of Spontaneous Preterm Delivery in a Danish/Norwegian Genome-Wide Association Study
Source: PLoS One. 2013 Apr 16;8(4):e61781. doi: 10.1371/journal.pone.0061781 (PMC3628886; doi:10.1371/journal.pone.0061781)
Supplement: Table S3 — Sex-stratified analysis, males, p<1.00×10^−3^. (DOCX) [file pone.0061781.s004.docx]

**Table S3. Sex-stratified analysis, males, p<1.00x10^-3^**

|  |  |  | Moba | | DNBC | | Combined analysis | | |
| --- | --- | --- | --- | --- | --- | --- | --- | --- | --- |
| Gene | SNP | Alleles | MAF | RR (95% CI) | MAF | RR (95% CI) | RR | p RR | p overall |
| IL1RAPL2 | rs6652393 | a/G | 0.40 | 1.32 (1.15, 1.52) | 0.38 | 1.14 (1.04, 1.26) | 1.20 (1.11, 1.29) | 5.47E-06 | 1.15E-05 |
|  | rs3008952 | a/G | 0.05 | 1.68 (1.33, 2.12) | 0.07 | 1.10 (0.92, 1.30) | 1.28 (1.11, 1.46) | 4.60E-04 | 7.50E-05 |
|  | rs2961403 | a/G | 0.06 | 1.66 (1.31, 2.10) | 0.07 | 1.10 (0.92, 1.30) | 1.27 (1.11, 1.46) | 5.46E-04 | 1.03E-04 |
|  | rs3131391 | A/g* | 0.29 | 1.20 (1.03, 1.39) | 0.31 | 1.18 (1.07, 1.31) | 1.19 (1.10, 1.29) | 3.16E-05 | 2.23E-04 |
| IL1RAPL2 | rs5962953 | A/g | 0.44 | 1.27 (1.10, 1.45) | 0.42 | 1.11 (1.01, 1.22) | 1.16 (1.07, 1.25) | 2.53E-04 | 4.37E-04 |
| IL1RAPL2 | rs2392623 | a/G | 0.46 | 0.82 (0.71, 0.94) | 0.45 | 0.88 (0.80, 0.97) | 0.86 (0.79, 0.93) | 1.85E-04 | 6.15E-04 |
| DDX26B | rs6528251 | A/g | 0.03 | 0.98 (0.65, 1.46) | 0.04 | 0.52 (0.35, 0.76) | 0.70 (0.53, 0.93) | 1.21E-02 | 8.09E-04 |
| IL1RAPL2 | rs6616564 | a/C | 0.46 | 0.82 (0.71, 0.95) | 0.45 | 0.88 (0.80, 0.97) | 0.86 (0.80, 0.93) | 2.33E-04 | 8.28E-04 |
| DDX26B | rs5974536 | A/c | 0.03 | 1.00 (0.66, 1.49) | 0.04 | 0.52 (0.35, 0.76) | 0.71 (0.54, 0.93) | 1.46E-02 | 8.77E-04 |
|  | rs1143856 | A/c | 0.22 | 1.11 (0.94, 1.31) | 0.16 | 1.24 (1.10, 1.39) | 1.19 (1.09, 1.31) | 1.86E-04 | 9.70E-04 |

*Deviates from HWE in Danish sample
