# Supplementary material for: X-Chromosomal Maternal and Fetal SNPs and the Risk of Spontaneous Preterm Delivery in a Danish/Norwegian Genome-Wide Association Study
Source: PLoS One. 2013 Apr 16;8(4):e61781. doi: 10.1371/journal.pone.0061781 (PMC3628886; doi:10.1371/journal.pone.0061781)
Supplement: Table S4 — Sex-stratified analysis, females, p<1.00×10^−3^. (DOCX) [file pone.0061781.s005.docx]

**Table S4. Sex-stratified analysis, females, p<1.00x10^-3^**

|  |  |  | MoBa | | DNBC | | Combined analysis | | |
| --- | --- | --- | --- | --- | --- | --- | --- | --- | --- |
| Gene | SNP | Alleles | MAF | RR | MAF | RR | RR | p RR | p overall |
| DACH2 | rs761202 | A/g | 0.20 | 1.80 (1.39, 2.32) | 0.20 | 0.89 (0.73, 1.09) | 1.17 (1.00, 1.36) | 4.96E-02 | 3.05E-05 |
| DACH2 | rs724620 | A/g | 0.20 | 1.78 (1.37, 2.28) | 0.21 | 0.88 (0.72, 1.07) | 1.15 (0.99, 1.35) | 6.70E-02 | 3.28E-05 |
| GRIA3 | rs5958198 | a/C | 0.37 | 1.06 (0.84, 1.33) | 0.33 | 1.45 (1.23, 1.71) | 1.30 (1.14, 1.49) | 9.01E-05 | 8.77E-05 |
| GRIA3 | rs6608062 | A/g | 0.37 | 1.01 (0.80, 1.27) | 0.33 | 1.44 (1.22, 1.69) | 1.28 (1.12, 1.46) | 2.40E-04 | 1.79E-04 |
| H2BFM | rs1024325 | A/g | 0.35 | 0.97 (0.77, 1.23) | 0.35 | 0.70 (0.58, 0.83) | 0.78 (0.68, 0.90) | 5.14E-04 | 3.65E-04 |
| DACH2 | rs4828153 | a/G | 0.17 | 1.76 (1.35, 2.30) | 0.16 | 1.00 (0.81, 1.24) | 1.25 (1.06, 1.48) | 7.35E-03 | 4.48E-04 |
| DACH2 | rs1291763 | a/G | 0.17 | 1.76 (1.35, 2.30) | 0.16 | 1.00 (0.80, 1.24) | 1.25 (1.06, 1.47) | 7.97E-03 | 4.52E-04 |
|  | rs7067173 | A/g | 0.33 | 1.00 (0.79, 1.27) | 0.34 | 0.69 (0.58, 0.82) | 0.79 (0.69, 0.91) | 8.96E-04 | 4.52E-04 |
|  | rs6636820* | a/G | 0.45 | 1.14 (0.91, 1.43) | 0.43 | 1.36 (1.16, 1.59) | 1.28 (1.13, 1.46) | 1.40E-04 | 4.54E-04 |
| H2BFWT | rs516045 | a/G | 0.35 | 0.97 (0.77, 1.23) | 0.35 | 0.70 (0.59, 0.83) | 0.79 (0.68, 0.90) | 6.08E-04 | 4.75E-04 |
|  | rs408278 | a/G | 0.33 | 1.01 (0.80, 1.29) | 0.34 | 0.70 (0.58, 0.83) | 0.80 (0.69, 0.91) | 1.32E-03 | 5.32E-04 |
| GRIA3 | rs4825847 | A/c | 0.37 | 1.06 (0.84, 1.33) | 0.34 | 1.40 (1.18, 1.64) | 1.27 (1.12, 1.45) | 3.13E-04 | 5.70E-04 |
| IL1RAPL1 | rs5928349 | A/g | 0.42 | 1.09 (0.86, 1.35) | 0.42 | 0.72 (0.61, 0.85) | 0.83 (0.73, 0.95) | 6.44E-03 | 6.29E-04 |
| DACH2 | rs5923575 | a/G | 0.44 | 0.65 (0.52, 0.82) | 0.38 | 1.11 (0.94, 1.30) | 0.93 (0.82, 1.06) | 2.92E-01 | 7.91E-04 |
|  | rs5945837 | A/g | 0.34 | 1.01 (0.79, 1.27) | 0.36 | 0.71 (0.59, 0.84) | 0.80 (0.70, 0.92) | 1.49E-03 | 8.51E-04 |
|  | rs12391099 | a/G | 0.08 | 0.60 (0.37, 0.95) | 0.05 | 1.58 (1.16, 2.14) | 1.18 (0.92, 1.52) | 1.87E-01 | 9.63E-04 |
| GRIA3 | rs983007 | a/G | 0.40 | 1.08 (0.86, 1.36) | 0.38 | 1.36 (1.16, 1.60) | 1.26 (1.11, 1.44) | 4.92E-04 | 9.94E-04 |
